# Osteoblasts secrete miRNA-containing extracellular vesicles that enhance expansion of human umbilical cord blood cells

**DOI:** 10.1038/srep32034

**Published:** 2016-09-02

**Authors:** Jess Morhayim, Jeroen van de Peppel, Eric Braakman, Elwin W. J. C. Rombouts, Mariette N. D. ter Borg, Amel Dudakovic, Hideki Chiba, Bram C. J. van der Eerden, Marc H. Raaijmakers, Andre J. van Wijnen, Jan J. Cornelissen, Johannes P. van Leeuwen

**Affiliations:** 1 Department of Internal Medicine, Erasmus Medical Center, Rotterdam, the Netherlands; 2 Department of Hematology, Erasmus Medical Center, Rotterdam, the Netherlands; 3 Department of Orthopedic Surgery, Mayo Clinic, Rochester, MN, USA; 4 Fukushima Medical University School of Medicine, Hikarigaoka, 960-1295, Fukushima, Japan

## Abstract

Osteolineage cells represent one of the critical bone marrow niche components that support maintenance of hematopoietic stem and progenitor cells (HSPCs). Recent studies demonstrate that extracellular vesicles (EVs) regulate stem cell development via horizontal transfer of bioactive cargo, including microRNAs (miRNAs). Using next-generation sequencing we show that human osteoblast-derived EVs contain highly abundant miRNAs specifically enriched in EVs, including critical regulators of hematopoietic proliferation (e.g., miR-29a). EV treatment of human umbilical cord blood-derived CD34^+^ HSPCs alters the expression of candidate miRNA targets, such as *HBP1*, *BCL2* and *PTEN*. Furthermore, EVs enhance proliferation of CD34^+^ cells and their immature subsets in growth factor-driven *ex vivo* expansion cultures. Importantly, EV-expanded cells retain their differentiation capacity *in vitro* and successfully engraft *in vivo*. These discoveries reveal a novel osteoblast-derived EV-mediated mechanism for regulation of HSPC proliferation and warrant consideration of EV-miRNAs for the development of expansion strategies to treat hematological disorders.

Extracellular vesicles (EVs) are secreted nano-sized cellular compartments that carry a specific biochemical cargo encompassing bioactive proteins, lipids and nucleic acids to regulate the function of recipient cells^1^,^2^,^3^. Circulating microRNAs (miRNAs), which are short non-coding RNAs of 21–25 nucleotides in length, are present in EVs and function as potent post-transcriptional regulators of gene expression^4^,^5^,^6^. EV-mediated miRNA transfer regulates various fundamental biological processes, including cell differentiation, proliferation and apoptosis^7^,^8^,^9^,^10^. Recent studies indicate that EV-miRNAs have biological roles in the hematopoietic system indicating the importance of EV-mediated paracrine signalling in hematopoiesis^11^,^12^.

Hematopoietic stem cells (HSCs) are multipotent cells responsible for constant blood supply by undergoing tightly regulated self-renewal, proliferation and differentiation into different mature blood cell types. In adult humans, hematopoiesis mainly occurs in the bone marrow niche, which provides a supportive network of cells that orchestrate HSC fate^13^,^14^. Osteolineage cells, ranging from primitive mesenchymal cells to bone-forming mature osteoblasts, are thought to be important to maintain hematopoietic stem and progenitor cells (HSPCs)^15^,^16^,^17^,^18^,^19^. The molecular mechanisms that control the crosstalk between osteolineage cells and HSPCs in humans remain largely unexplored. Comparative gene expression profiling identified a number of molecules ranging from adhesion molecules to secreted factors, such as growth factors and cytokines, which may be essential for hematopoietic activity^20^,^21^.

Even though there is no direct evidence yet, EVs may participate in the regulation of HSPC maintenance. We previously reported the protein content of human osteoblast-derived EVs at different stages of differentiation and mineralization^22^. Beyond protein cargo, it has become increasingly clear that miRNAs play a pivotal role in the regulation of HSPC fate^23^,^24^,^25^. Therefore, characterization of the miRNA content of osteoblast-EVs is necessary for appreciating the complexity of the HSPC-osteolineage-cell crosstalk and may open new avenues for clinical applications.

In the present study, we elucidate the miRNA profile of EVs secreted from human pre-osteoblasts using next-generation sequencing. Based on *in silico* target prediction analyses and *in vitro* biochemical analyses we define candidate hematopoietic development pathways affected by osteoblast-EVs. Importantly, we investigate the potency of osteoblast-EVs to promote *ex vivo* expansion of umbilical cord blood (UCB)-derived CD34^+^ HSPCs. We further verify the functionality of the expanded cells *in vivo* by performing xenogeneic transplantation in immunodeficient mice. Our findings provide a foundation for the utilization of EVs as novel tools to modulate hematopoiesis for the development of suitable strategies to treat hematological disorders.

## Results

### Human osteoblasts secrete EVs that contain small RNAs

**Figure 1:**
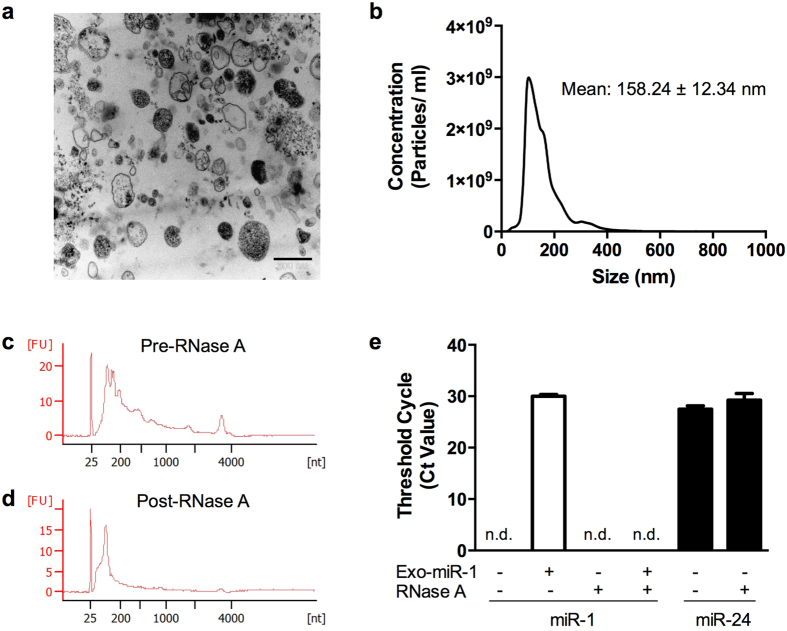
Characterization of osteoblast-derived EVs and the RNA inside EVs. **(a)** Representative transmission electron microscope image (×28,000) of EVs isolated from human osteoblasts. Scale bar: 500 nm. **(b)** Nanoparticle tracking analysis shows EV size distribution and concentration. (N = 3). **(c,d)** Representative Agilent 2100 Bioanalyzer (Pico) RNA profiles of osteoblast-EVs (**c**) before and (**d**) after RNase A treatment (100 ng/ml, 30 minutes at 37 °C). FU, fluorescent units. (N = 3). **(e)** Quantification of vesicular human miR-24 and miR-1 levels by qPCR in the presence or absence of exogenous synthetic miR-1 and RNase A. Data is presented as raw threshold cycle numbers (Ct values) (mean ± SD) (N = 3). n.d. denotes Ct values above 35 or not detectable.

To characterize osteoblast-derived EVs, SV-HFO cells were cultured for 12–14 days, and EVs were isolated from the conditioned medium by a series of ultracentrifugation steps. Transmission electron microscopy (Fig. 1a) and nanoparticle tracking analysis (Fig. 1b) show the heterogeneous morphology of the EV population with an average size of 158 nm. Agilent Bioanalyzer RNA profiles show that osteoblast-EVs lack the typical cellular rRNAs, and instead are enriched with small RNAs (Fig. 1c). The EV-RNA peak is retained when the EVs are treated with RNase A prior to RNA isolation (Fig. 1d), verifying that the majority of the detected RNA is indeed present inside the EVs.

To demonstrate that small EV-RNAs comprise miRNAs, we performed quantitative real-time PCR (qPCR) of the widely expressed human miR-1 and miR-24. As shown in Fig. 1e, osteoblast-EVs are devoid of miR-1 (*left panel*) while they contain relatively high amounts of miR-24 (*right panel*). Interestingly, RNase A treatment does not significantly alter the miR-24 level, confirming the presence of nuclease-resistant miR-24 inside the EVs. In contrast, exogenously added synthetic miR-1 was immediately degraded when spiked into the sample. Collectively, these data demonstrate that miRNAs are present in the heterogeneous population of osteoblast-EVs.

### Osteoblast-EVs contain a specific set of abundant miRNAs

**Figure 2:**
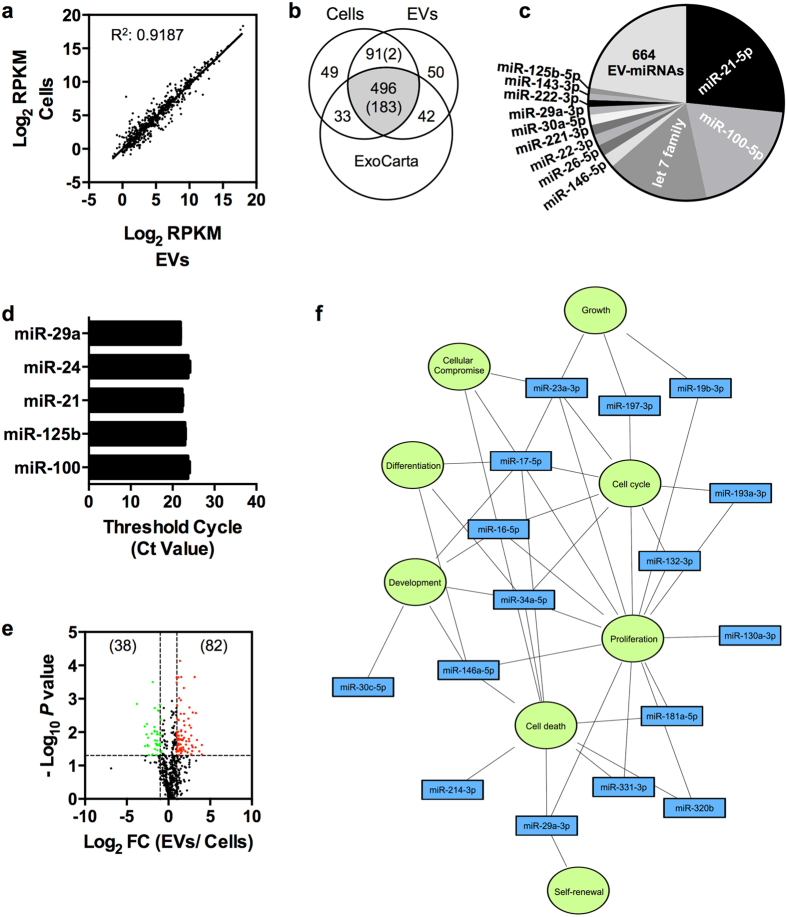
Next-generation sequencing miRNA profiling of osteoblast-EVs. **(a)** Scatter plot shows the strong correlation (R^2^: 0.9187) of normalized read counts (average RPKM) between cellular and vesicular miRNAs (N = 3). **(b)** Venn diagram shows the number of miRNAs detected in osteoblasts and EVs in comparison with ExoCarta. Numbers in brackets denote the number of highly abundant miRNAs with reads greater than 100 RPKM among all EV miRNAs. **(c)** Pie chart shows the normalized read count proportions of all EV-miRNAs. Only the top 15 most abundant EV-miRNAs, including let-7f-5p, let-7i-5p, let-7g-5p and let-7a-5p grouped as let7 family, are displayed. **(d)** Validation of highly abundant EV miRNAs by TaqMan qPCR miRNA assay. Data is presented as raw Ct values (mean ± SD) (N = 3). **(e)** Volcano plot (significance versus fold change) shows the significantly (*P* < 0.05, compared with osteoblasts by Student’s *t*-test) abundant (≥2-fold; red) and depleted (≤0.5-fold; green) miRNAs in EVs. Numbers in brackets denote the number of differentially expressed miRNAs. FC, fold change. **(f)** IPA network map shows the predicted biological functions significantly targeted by some of the highly abundant (RPKM >100) enriched EV miRNAs. Blue, EV miRNAs; Green, biological functions.

To evaluate the miRNA profile of osteoblast-EVs, we performed next-generation sequencing using total RNA from three independent osteoblast cultures and their corresponding EVs. The majority of the miRNAs yielded similar reads counts in cells and EVs (Fig. 2a). In total, we identified 761 mature miRNAs, of which 496 miRNAs are shared between cells and EVs and listed in ExoCarta (Fig. 2b)^26^,^27^. Notably, all miRNAs exclusively present in cells (82 miRNAs) or EVs (92 miRNAs) are typically of low abundance. Only 185 EV-miRNAs are significantly abundant with levels above 100 normalized reads per million. This set encompasses 183 miRNAs that are among the 496 common miRNAs in both cells and EVs, as well as 2 miRNAs that are not yet listed in ExoCarta. The top 15 of miRNAs present in EVs produce ~75% of all the EV-miRNA reads, and more than half of the reads belong to just three major miRNA families related to miR-21-5p, miR-100-5p and let-7 (let-7f-5p, let-7i-5p, let-7g-5p, let-7a-5p) (Fig. 2c). Figure 2d shows the qPCR validation of selected abundant EV-miRNAs.

Next, we performed relative quantitation of cellular and vesicular miRNAs to assess whether a unique set of miRNAs is enriched in EVs. The Volcano plot in Fig. 2e shows all EV-miRNAs with statistical significance (*P* < 0.05) and differential abundance (≥two-fold enrichment); the low abundance miRNAs unique to EVs and cells were excluded from this analysis. The results essentially reveal that EVs are preferentially loaded with 82 miRNAs (red dots) and depleted of 38 miRNAs (green dots). Table 1 shows the highly abundant and enriched 33 miRNAs with normalized reads above 100 (Supplementary Fig. S1). Among these miRNAs, miR-146a-5p and miR-29a-3p are the most abundant, while miR-1246 and miR-1290 are most enriched (>10-fold) in EVs. Interestingly, 21 out of 33 enriched EV-miRNAs have not been previously reported as EV markers that are most commonly detected across all tissues and body fluids^28^. Hence, these 21 miRNAs may reflect selective sorting into osteoblast-EVs.

Ingenuity Pathway Analysis (IPA) was performed to predict the impact of enriched EV-miRNAs on target cell gene expression and phenotype. The most significantly annotated molecular functions are cellular development, cellular growth and proliferation, cell cycle, cell death and survival and cellular compromise (Fig. 2f). Together, our comparative analyses revealed overrepresentation of a selective group of miRNAs in osteoblast-EVs, indicating specific packaging of biologically functional regulatory molecules as EV cargo.

### Osteoblast-EVs are enriched with miRNAs crucial for hematopoiesis

**Figure 3:**
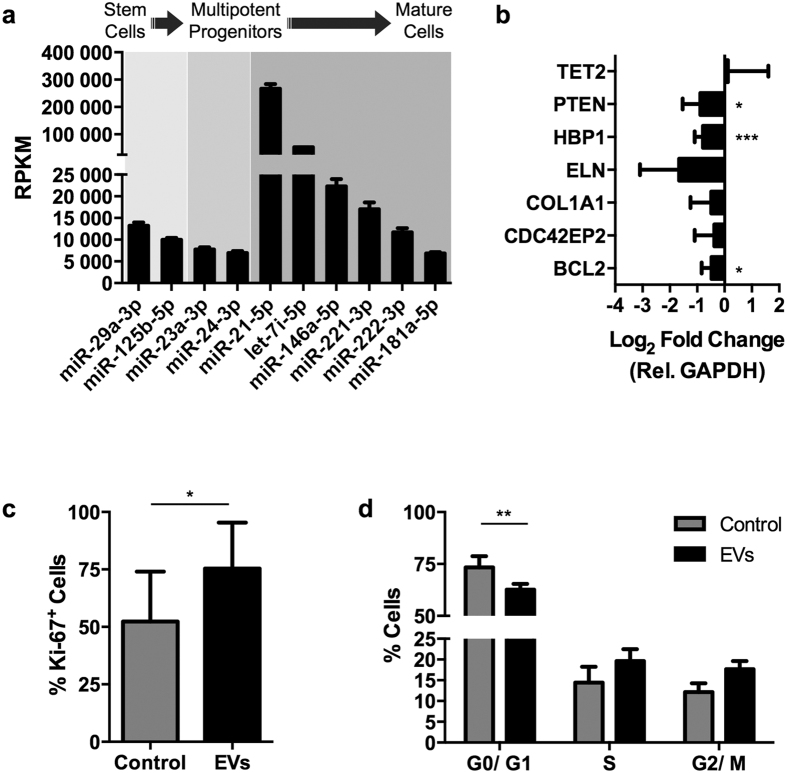
Osteoblast-EVs down-regulate miR-29a target genes and stimulate proliferation and cell cycle progression of UCB-derived CD34^+^ cells. (**a**) RPKM values (mean ± SD) of osteoblast-EV miRNAs known to be involved in different phases of hematopoiesis. (**b**) Expression levels (mean ± SD) of candidate mRNA targets of miR-29a in human UCB-derived CD34^+^ cells after 24 hours of incubation in GBGM supplemented with SCF and Flt3L in the absence (control) or presence of osteoblast-EVs (N = 5). *P < 0.05, ***P < 0.005, compared with control by Student’s *t*-test. (**c**) Proliferation assay, determined by the % of Ki-67^+^ CD34^+^ cells (mean ± SD), after 24 hours incubation in the absence (control) or presence of osteoblast-EVs (N = 3). *P < 0.05, compared with control by Student’s *t*-test. (**d**) Cell cycle distribution, using propidium iodide (PI) staining of CD34^+^ cells (mean ± SD), after 24 hours incubation in the absence (control) or presence of osteoblast-EVs (N = 3). **P < 0.01, compared with control by two-way ANOVA.

Each stage of hematopoietic differentiation is characterized by a specific miRNA signature^29^. We analyzed the osteoblast-EV-miRNA content for known regulators of hematopoiesis to find cues for EV-mediated HSPC-osteolineage-cell crosstalk. Indeed, a number of miRNAs that may control distinct stages of hematopoietic development are detected in high abundance in EVs (Fig. 3a). Hence, we investigated whether treatment of HSPCs with osteoblast-EVs would alter expression of specific mRNAs that are targeted by miRNAs enriched in osteoblast-EVs.

We focused initially on miR-29a, which is significantly enriched in osteoblast-EVs, because it is known to be involved in early stages of hematopoiesis as a regulator of self-renewal, survival and proliferation of HSCs and HSPCs^30^. *In silico* target prediction analysis by TargetScan combined with supervised literature searches refined the large list of potential targets to a defined list of validated miR-29a target genes relevant to HSPCs. By qPCR analysis we evaluated the expression level of miR-29a target genes involved in proliferation (*TET2*, *PTEN*), apoptosis (*BCL2*), cell cycle regulation (*HBP1*, *CDC42EP2*) and extracellular matrix adhesion (*COL1A1*, *ELN*) of HSPCs^30^,^31^,^32^. Osteoblast-EVs reduce the expression of all selected target genes, except *TET2*, in human UCB-derived CD34^+^ HSPCs (Fig. 3b). EVs significantly down-regulate the expression of *HBP1*, *BCL2* and *PTEN*, suggesting that osteoblast-EVs control proliferation of the recipient HSPCs. Proliferation assays that monitor Ki-67 staining (Fig. 3c) and cell cycle analysis based on DNA content (Fig. 3d) establish that osteoblast-EVs stimulate proliferation and cell cycle progression of the CD34^+^ cells 24 hours after treatment. Thus, the integrated results of both *in silico* and *in vitro* approaches indicate that osteoblast-EVs are enriched with miRNAs involved in signaling cascades that regulate HSPC proliferation.

### Osteoblast-EVs promote *ex vivo* expansion of CD34^+^ HSPCs

**Figure 4:**
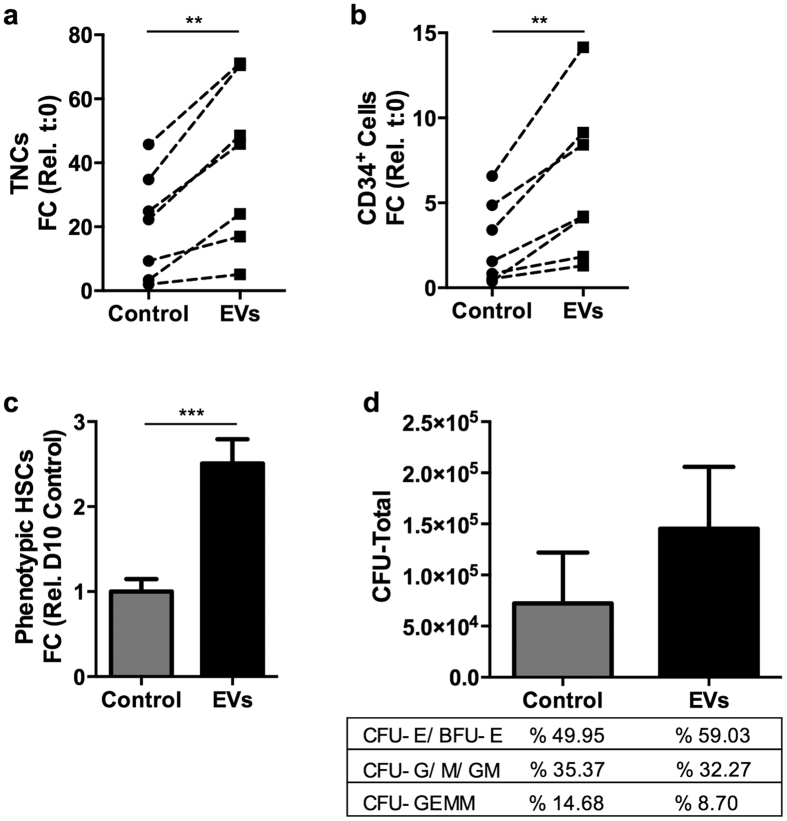
Osteoblast-EVs enhance *ex vivo* expansion of CD34^+^ cells. (**a,b)** Osteoblast-EVs increase the expansion of (**a**) total nucleated cells (TNCs) and (**b**) CD34^+^ cells after 10 days of expansion with SCF and Flt3L compared to cells cultured in the absence of EVs (control) (N = 7). Expansion is shown as fold change (FC) increase in total cell number compared to input. **P < 0.01, compared with control by Student’s *t*-test. **(c)** Osteoblast-EVs increase the number (mean ± SD) of phenotypic HSCs (lin^−^ CD34^+^ CD38^low^ CD45RA^low^ CD90^+^) compared to control on day 10 (N = 3). ***P < 0.005, compared with control by Student’s *t*-test. **(d)** EV-expanded immature UCB cells retain their differentiation capacity in colony forming unit (CFU) assay (mean ± SD) (N = 3). The frequencies of the myeloid and erythroid lineages are shown in the table.

We evaluated the capacity of osteoblast-EVs to promote the *ex vivo* expansion of human UCB-derived CD34^+^ cells in growth factor (stem cell factor, SCF and Fms-related tyrosine kinase 3 ligand, Flt3L)-driven serum-free expansion cultures. Osteoblast-EVs induce a two-fold expansion of both total number of viable nucleated cells (TNCs) (*P* < 0.01) and CD34^+^ cells (*P* < 0.01) in 10 days as compared to control cultures (Fig. 4a,b). We also assessed the presence of the most immature CD34^+^ cell subset in the expanded cells by multicolor flow cytometry using markers for primitive HSPCs (lin^-^ CD34^+^ CD38^low^ CD45RA^low^ CD90^+^), referred to as phenotypic HSCs. EV treatment also increases the number of phenotypic HSCs more than two-fold (*P* < 0.005) compared to the control on day 10 (Fig. 4c). Interestingly, the expansion potential of EVs remains clearly evident even in the presence of other highly effective expansion factors, such as StemRegenin 1 (SR1) and thrombopoietin (TPO)^33^,^34^ (Supplementary Fig. S2).

Next, we investigated whether the CD34^+^ cells that were expanded *ex vivo* using EVs retain their differentiation capacity *in vitro* by performing colony-forming unit (CFU) assay. EV-expanded cells exhibit a higher clonogenicity, most likely due to the increased number of viable and functional CD34^+^ cells after expansion (Fig. 4d). However, the frequencies of multilineage progenitors (CFU-GEMM), erythroid progenitors (CFU-E/BFU-E) and granulocyte/macrophage progenitors (CFU-G/M/GM) remain comparable to the control, indicating that EVs promote expansion but do not favor specific hematopoietic lineages. These findings demonstrate the potency of osteoblast-EVs to promote growth factor-driven HSPC expansion while retaining the pool of progenitor cells that give rise to erythrocytes and myeloid cells *in vitro*.

### Osteoblast-EV-expanded cells retain *in vivo* engraftment potential

**Figure 5:**
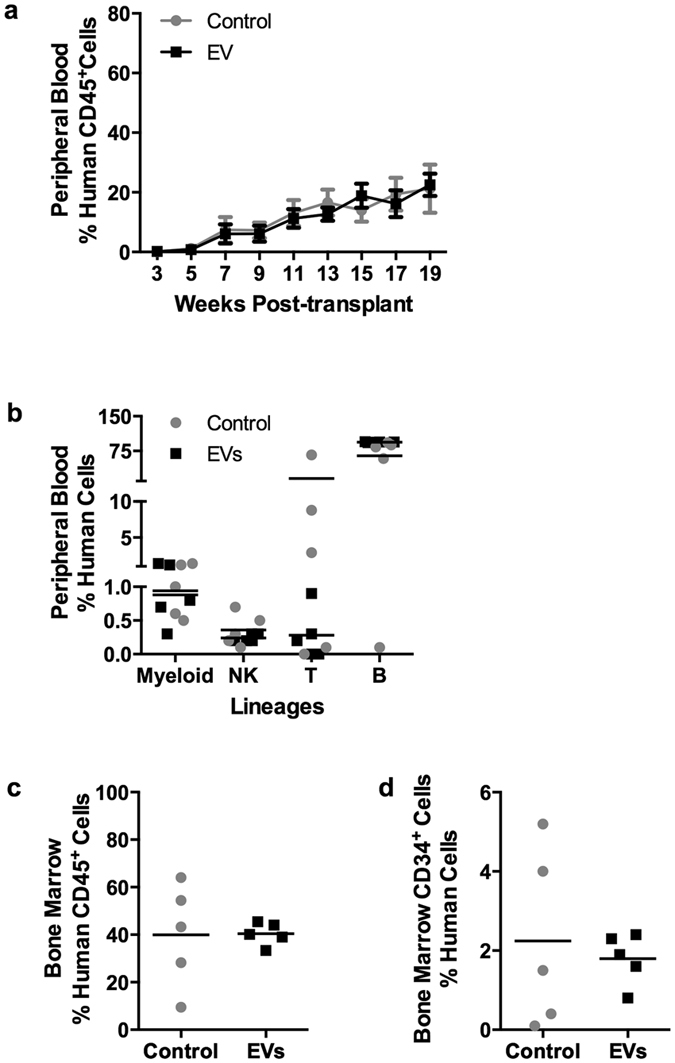
EV-expanded CD34^+^ cells successfully engraft and re-populate NSG mice. **(a)** Human chimerism level (% human CD45^+^ cells) (mean ± SD) in peripheral blood at several time-points after transplantation of CD34^+^ cells expanded in the absence (control) and presence of EVs (N = 5 mice/group). (**b**) The frequencies of human lymphoid and myeloid lineages in peripheral blood after 19 weeks. **(c)** Human chimerism level in the bone marrow after 21 weeks. C_V_ (coefficient of variation) is 54.13% and 11.74% for control and EVs, respectively. **(d)** The frequencies of human CD34^+^ progenitors within the human CD45^+^ fraction in the bone marrow after 21 weeks. C_V_ is 100.75% and 35.79% for control and EVs, respectively.

To assess the impact on engraftment and hematopoietic repopulating ability of the *ex vivo* expanded cells, sublethally irradiated immunodeficient NOD.Cg-*Prkdc*^*scid*^ *Il2rg*^*tm1Wjl*^/SzJ (NSG) mice were transplanted with CD34^+^ progeny cells derived from 10^5^ seeded cells after expansion with or without EVs for 10 days. Engraftment was defined as at least 0.1% human chimerism in the peripheral blood. All mice show similar levels of human chimerism (~20%) in both control and EV treatment groups at 19 weeks after transplantation (Fig. 5a). EV treatment does not alter the speed and quality of recovery of different human lymphoid and myeloid lineages in NSG mice. The predominant human cell population after engraftment consists of CD19^+^ B-cells, as well as very low levels of other lymphoid (NK and T-cells) and myeloid cells (Fig. 5b). Moreover, both control and the EV treatment groups exhibit similar levels of CD45^+^ human chimerism in recipient bone marrow at 21 weeks after transplantation (Fig. 5c). Similar to the peripheral blood, the majority of human cells in the bone marrow expressed the B-cell marker CD19 (Supplementary Fig. S3). As expected, only a small (2%) fraction of human cells is positive for CD34 (Fig. 5d). We note that the mice in the control group show a high degree of variability (C_V_: 54.13%, Fig. 5c; C_V_: 100.75%, Fig. 5d) of chimerism in the bone marrow, possibly explained by the lower number of total transplanted cells after expansion. Taken together, these findings clearly demonstrate that *ex vivo* EV treatment retains the engraftment potential of human cells in NSG mice.

### Osteoblast-EVs stimulate the proliferation of immature cells

**Figure 6:**
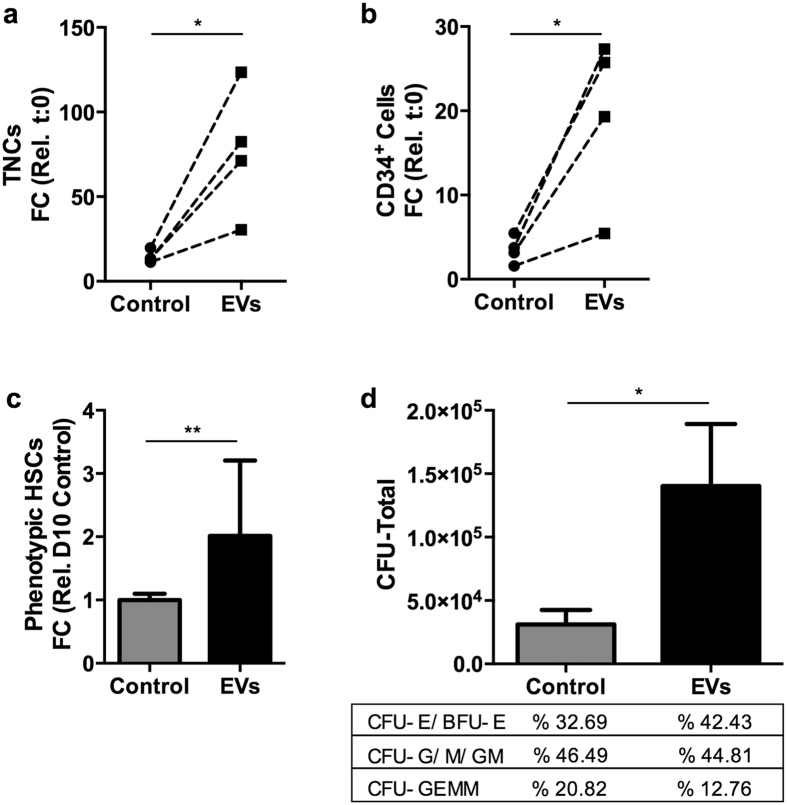
Osteoblast-EVs enhance *ex vivo* expansion of sorted phenotypic HSCs. (**a,b)** Osteoblast-EVs improve the expansion capacity of (**a**) total nucleated cells (TNCs) and (**b**) CD34^+^ progenitors after 10 days of expansion with SCF and Flt3L compared to cells cultured in the absence of EVs (control) (N = 4). Expansion is shown as fold change increase in total cell number compared to input. *P < 0.05, compared with control by Student’s *t*-test. **(c)** Osteoblast-EVs increase the number (mean ± SD) of phenotypic HSCs compared to control on day 10 (N = 4). **P < 0.01, compared with control by Student’s *t*-test. **(d)** EV-expanded immature UCB cells retain their differentiation capacity in CFU assay (mean ± SD) (N = 3). The frequencies of the myeloid and erythroid lineages are shown in the table. *P < 0.05, compared with control by Student’s *t*-test.

Most conventional expansion protocols, which provide short-term robust proliferation of the CD34^+^ progenitor cells, are accompanied by concomitant differentiation and result in loss of primitive HSC sub-populations^35^,^36^,^37^. To study the effect of EVs on immature stem cells, we sorted phenotypic HSCs as a starting population. Osteoblast-EV treatment of phenotypic HSCs significantly induces the expansion of TNCs (Fig. 6a), CD34^+^ cells (Fig. 6b) and phenotypic HSCs (Fig. 6c) after 10 days. Corroborating these results, CFU assays reveal an increase in the number of the immature cells while retaining the frequency of the different lineages (Fig. 6d).

**Figure 7:**
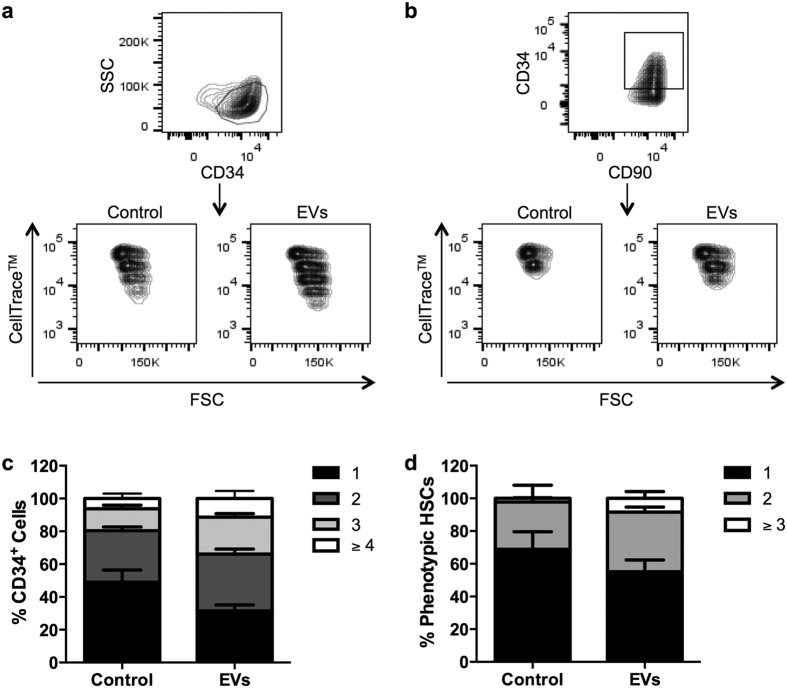
Osteoblast-EVs stimulate the proliferation of the immature cells. **(a,b)** Sorted phenotypic HSCs were loaded with CellTrace^TM^ Violet and incubated in the absence (control) or presence of osteoblast-EVs for 5 days. Flow cytometry plots show the distribution of the progeny of (**a**) CD34^+^ progenitors and (**b**) CD90^+^ phenotypic HSCs. **(c,d)** The percentages (mean ± SD) of cells that have divided 1–4 times within (**c**) CD34^+^ progenitors and (**d**) CD90^+^ phenotypic HSCs (N = 2).

To determine the effect of osteoblast-EVs on the maintenance of CD34 and CD90 expression after successive cell divisions of phenotypic HSCs, we used CellTrace^TM^ Violet staining. Cells that were grown in the presence of osteoblast-EVs undergo a higher number of cell divisions while keeping their stem cell phenotype after 5 days compared to control (Fig. 7a,b). Figure 7c,d show the fraction of cells that have divided up to 4 times within the CD34^+^ cell population in the absence or presence of EVs. Our data show that EVs stimulate the cell division kinetics of the immature cells, resulting in an increased number of CD34^+^ cells.

## Discussion

EV-mediated intercellular communication is an exciting new area of research that is rapidly evolving thanks to the emergence of powerful tools that enable characterization of their bioactive cargo. We used next-generation sequencing to study the miRNA profile of human osteoblast-derived EVs. Based on the overrepresented EV-miRNAs we delineated the targeted biological functions associated with hematopoiesis and verified *in silico* predictions with *in vitro* results. We show that osteoblast-EVs stimulate the proliferation of UCB-derived CD34^+^ HSPCs and subsequent expansion of functional primitive cells, which retain their multipotency *in vitro* and successfully engraft *in vivo*. Our findings provide a fundamental description of the biological roles of EVs in mediating crosstalk between osteolineage cells and HSPCs. Equally important, osteoblast-derived EVs may present potential value in clinical applications to treat hematopoietic disorders.

There is considerable interest in understanding how circulating miRNAs exert their effect in a paracrine manner via EV-mediated delivery to the target tissue. We performed high throughput analysis of miRNAs to assess whether human osteoblast-EVs utilize small RNAs to communicate with HSPCs in the bone marrow niche. As expected, the EV-miRNA profile mirrors that of the osteoblastic donor cell, but we also detected a large number of miRNAs that appear to be enriched in EVs. Importantly, we identified a number of miRNAs that are abundant in osteoblast-EVs and are known regulators of hematopoiesis. To predict targeted pathways, we focused on miR-29a because it controls early steps of hematopoiesis and is enriched in EVs compared to the donor cells. Our study shows that osteoblast-EVs are capable of down-regulating the expression of cell cycle- and growth-related miR-29a target genes in CD34^+^ cells. We further demonstrate that osteoblast-EVs stimulate proliferation and cell cycle progression of CD34^+^ cells, in accordance with the expression profile of down-regulated target genes. Further investigation is required to elucidate the precise mechanism of EV action by showing a direct evidence of delivery of miR-29a via EVs, followed by miR-29a regulation of the targets. Nevertheless, attributing the proliferative effect of EVs solemnly to one miRNA would be oversimplification of the complexity of EV-mediated cell-to-cell communication. In fact, our previous studies reporting the discovery of osteoblast-EV proteins capable of stimulating cell growth demonstrates the necessity of carefully investigating other functional cargo to understand the EV effect.

Stem cells are special cells with remarkable regenerative abilities, which make them very attractive for the development of cell-based therapies. A complex network of environmental cues tightly regulates stem cell fate determination, including self-renewal and differentiation. Ratajczak and colleagues were one of the firsts to report the role of EVs in stem cell regulation^38^. They showed that embryonic stem cells released EVs containing regulatory proteins and mRNA capable of reprogramming HSCs. Since then numerous studies reported the role of EVs in stem cell biology with a major focus on miRNA transfer, suggesting a critical paracrine role for EVs in stem cell niches^39^,^40^,^41^,^42^. We previously showed that osteoblast-EVs induced proliferation of bone-metastasizing prostate cancer cells^22^. Recent studies indicate that prostate cancer cells compete with HSCs for the bone marrow niche, emphasizing the importance of EVs in the regulation of HSC growth and survival^43^. Hematopoietic differentiation is characterized by specific miRNA signatures, which act in each step of linage decision to ensure proper hematopoiesis^25^,^29^. The osteoblast-EV-miRNA profile we describe here provides further insights into the complexities of the HSPC-osteolineage-cell crosstalk.

The ability of osteoblast-derived EVs to regulate HSPCs brings exciting possibilities for therapeutic applications. UCB is an increasingly important source of HSPCs for the treatment of a variety of hematological disorders. However, UCB grafts contain a low number of HSPCs that poses as a limiting factor for proper engraftment, which leads to delayed hematopoietic recovery and patient morbidity and mortality^44^,^45^. Over the past few decades, studies have been focusing on the establishment of *ex vivo* culture systems to expand UCB-derived HSPCs for improved engraftment and post-transplantation recovery^33^,^36^. Therefore, we addressed whether we could therapeutically exploit the proliferation promoting capacity of osteoblast-EVs to expand UCB-derived CD34^+^ cells *ex vivo*. Conventional expansion protocols make use of a cocktail of growth factors to provide culture conditions optimized for HSPC expansion to obtain short-term robust proliferation^33^,^46^. Here, we limited the combination of growth factors to the minimum required potency to better study the EV effect. Our findings show that EVs significantly enhance the *ex vivo* expansion of CD34^+^ cells, including the most primitive subset of phenotypic HSC. Most importantly, EV-expanded CD34^+^ cells differentiate *in vitro* and successfully engraft and re-populate NSG mice *in vivo*. Further integrated genomic and proteomic analyses are of utmost importance to unravel candidate EV cargo components that can be modulated to increase expansion capacity that will meet the clinical need.

In conclusion, our findings provide a new paradigm for the role of EVs in the regulation of stem cell niches. We propose that EVs contain a signature miRNA profile that participates in the HSPC-osteolineage-cell crosstalk. It remains to be determined whether EV-miRNAs act in concert with other regulatory EV cargo, such as mRNAs and proteins, which may contribute to the proliferative effect of osteoblast-EVs. Identification of such critical EV components opens up avenues to be exploited clinically to develop novel approaches not only for the treatment of hematological disorders but in a broader context also for use in regenerative medicine.

## Methods

### Mice

*In vivo* studies were performed using young NSG mice (8 to 10 weeks old, female). All mice were handled under sterile conditions and housed in ventilated micro-isolation cages with filter tops. Mice were fed *ad libitum*. All animal experiments were performed in accordance with the Dutch law on Animal experiments and approved by the Committee on the Ethics of Animal Experiments of the Erasmus University Medical Center (approval number 3371), Rotterdam, the Netherlands.

### Cell culture

Simian virus 40-immortalized human osteoblast cells (SV-HFO cells) were seeded at a density of 5 × 10^3^ cells/cm^2^ and cultured in α-MEM (GIBCO, Paisley, UK) supplemented with 20 mM HEPES, pH 7.5 (Sigma, St. Louis, MO, USA), streptomycin/penicillin, 1.8 mM CaCl_2_ (Sigma), 10 mM β-glycerophosphate (Sigma) and 2% depleted (100,000 g for 90 minutes at 4 °C)-FCS (GIBCO) at 37 °C in a humidified atmosphere of 5% CO_2_ for 12–14 days. The culture medium was replaced every 2–3 days. SV-HFOs were washed with 1X PBS and refreshed with serum-free medium 24 hours prior to EV isolation. All experiments with human UCB were performed in accordance with the Dutch law on Medical Scientific Research with Humans and approved by the Medical Ethical Committee of the Erasmus University Medical Center (MEC-2009-410), Rotterdam, the Netherlands and written informed consent from the mothers was obtained prior to UCB donation. UCB was collected in several hospitals using Stemcare®/CB collection blood bag system (Fresenius Kabi Norge AS, Halden, Norway). Within 48 hours after collection, mononuclear cells were isolated using ficoll (Lymphoprep™, Fresenius Kabi Norge AS). CD34^+^ cells and viable DAPI^−^Lin^−^CD34^+^CD38^low^CD45RA^low^CD90^+^ cells were isolated as described previously^47^. 20,000 CD34^+^ cells and 10,000 CD34^+^CD90^+^ cells were cultured in serum-free Glycostem Basic Growth Medium (GBGM; Glycostem, Oss, the Netherlands) supplemented with SCF (50 ng/ ml, Cellgenix, Freiburg, Germany) and Flt3L (50 ng/ml, Cellgenix), with or without osteoblast-EVs at 37 °C in a humidified atmosphere of 5% CO_2_. Cells were refreshed every 2–3 days with fresh culture medium supplemented with newly isolated EVs. In some experiments SR1 (1 μM, Cellagen Technology, San Diego, CA, USA) and TPO (50 ng/ml, Cellgenix) were added. In some experiments, cells were labeled with CellTrace^TM^ Violet (Thermo Fisher Scientific, Waltham, MA, USA) according to the manufacturer’s instructions.

### EV isolation and characterization

Osteoblast-EVs were isolated from 20 ml conditioned medium by low speed centrifugation (1500 rpm, 5 minutes; 4500 rpm, 10 minutes) followed by ultracentrifugation (20,000 g, 30 minutes; 100,000 g, 1 hour at 4 °C) of the supernatant using the SW32Ti rotor (Beckman Coulter, Fullerton, CA, USA). EVs were prepared as 100 μl suspensions, and the amount of experimental EV dose was determined as 5% (v/v). Transmission electron microscopy images were taken as previously described^22^. EV size distribution and concentration was measured with NanoSight LM10 (Nanosight Ltd., Amesbury, UK) equipped with a 405 nm laser. Each sample was tracked for 60 seconds with 3 repetitions. The data was processed by NTA 2.3 software.

### RNA isolation and quantitative real-time PCR

The purified EV pellet was incubated with or without RNase A (100 mM) and synthetic miR-1 (20 pM) at 37 °C for 30 minutes, and total RNA was isolated using the TRIzol reagent (Thermo Fisher Scientific) according to the manufacturer’s instructions. RNA concentration was determined using Nanodrop (Thermo Fisher Scientific) and size distribution was checked on an Agilent Bioanalyzer RNA 6000 Pico chip (Thermo Fisher Scientific). RNA from CD34^+^ cells was isolated using NucleoSpin RNA XS kit (Macherey-Nagel, Duren, Germany) according to the manufacturer’s instructions. Quantitative real-time PCR for mRNAs and miRNAs were performed using the SYBR^®^ Green (Eurogentec, Seraing, Belgium) and Taqman^®^ kits (Thermo Fisher Scientific), respectively, according to the manufacturer’s instructions. The primer sequences are listed in Supplementary Table S1.

### Next-generation sequencing and bioinformatic analysis of miRNAs

Sequencing of miRNAs was performed by Illumina HiSeq with samples prepared with the NEBNext Small RNA library preparation kit. We used the CAP-miRSeq bioinformatic pipeline for mapping of miRNA reads data analysis^48^. In brief, adaptor sequences were removed from 50 bp reads using Cutadapt^49^ and sequences of sufficient size (>17 nucleotides in length) were aligned to the hg19 reference genome and miRBase 19 reference sequences using Bowtie^50^. Quantitative analysis of known and predicted miRNAs was performed using miRDeep2 51. Sequencing data are available in the ArrayExpress database (www.ebi.ac.uk/arrayexpress) under accession number E-MTAB-4560. Comparative analyses of the EV data were obtained by querying the ExoCarta (V5, 29 July 2015) database. IPA (Ingenuity® Systems, www.ingenuity.com) and TargetScan (Release 7.0) were used to predict target pathways and genes.

### Flow cytometry

Analysis of proliferation and cell cycle progression of CD34^+^ cells was performed using flow cytometry by monitoring the proliferation marker Ki-67 relative to DNA content. Cells were incubated with or without EVs for 24 hours, and stained with Alexa Fluor^®^ 488-conjugated Ki-67 antibody (BD Biosciences, San Jose, CA, USA) followed by the addition of propidium iodide (BD Biosciences) according to the manufacturers instructions. All samples were immediately analyzed using BD Accuri^TM^ C6 flow cytometry (BD Biosciences), and the data was analyzed using C6 software. Flow cytometry was also used to examine subpopulations of CD34^+^ cells. Absolute numbers of viable human CD34^+^ cells were determined by a single platform flow cytometric assay using anti-FITC-CD45, anti-CD34-PE, fluorescence reference counting beads (Beckman Coulter) and DAPI (Sigma). The frequencies of human phenotypic HSCs were determined using anti-Lin-FITC, anti-CD38-APC, anti-CD90-PE (eBiosciences, Vienna, Austria), anti-CD34-PE-Cy7, anti-CD45RA-APC-H7 (BD Biosciences); and DAPI. Human chimerism in mice blood and bone marrow was determined using anti-mouse CD45-eFluor450, (eBiosciences) and anti-human CD45-APC-Cy7 (BioLegend, London, UK). The human lymphoid and myeloid lineages were analyzed by staining with anti-CD19-PE, anti-CD3-APC, anti-CD33-PerCP-eFluor710, anti-CD56-PE-Cy7, and anti-CD15-BV510. All samples were analyzed using BD FACSCantoII (BD Biosciences), and the data was analyzed using FlowJo software (Tree Star, Inc., Ashland, OR, USA).

### CFU assays

Suspensions of 300 expanded CD34^+^ cells in MethoCult™ medium (MethoCult™ GF H4434, Stemcell Technologies, Vancouver, BC, Canada), were incubated in standard culture dishes for 11 days at 37 °C in a humidified atmosphere of 5% CO_2_. The colonies were counted using an inverted light microscope, and the different lineages were evaluated by morphology according to the manufacturer’s instructions (Stemcell Technologies).

### Transplantation

NSG mice were sub-lethally irradiated (3 Gy), and intravenously transplanted with the progeny generated from 10^5^ CD34^+^ cells cultured with or without EVs for 10 days. Peripheral blood was collected at 2 weeks intervals starting at 3 weeks post-transplantation through a small incision in the tail vein. At 21 weeks post-transplantation, the mice were sacrificed by cervical dislocation, and the bone marrow was harvested from the femurs as previously described^52^.

### Statistics

The results were described as mean ± SD based on at least two independent experiments performed with independent EV isolations and/ or different UCB donors. Significance was calculated using Student’s *t*-test and two-way ANOVA test, and *P* values < 0.05 were considered significant.

## Additional Information

**How to cite this article**: Morhayim, J. *et al*. Osteoblasts secrete miRNA-containing extracellular vesicles that enhance expansion of human umbilical cord blood cells. *Sci. Rep*. **6**, 32034; doi: 10.1038/srep32034 (2016).

## Supplementary Material

Supplementary Information

## Figures and Tables

**Table 1:** The list of highly abundant (RPKM >100) EV miRNAs significantly (*P* < 0.05) enriched (≥two-fold) in osteoblast-EVs compared to the cells.

Mature miRNAs	EVs (RPKM)	Cells (RPKM)	Fold Change	*P* value
miR-146a-5p	22222.93	8704.45	2.6	0.00007
miR-29a-3p	13142.2	6705.16	2.0	0.00023
miR-23a-3p	7703.92	1420.23	5.7	0.00183
miR-181a-5p	6769.81	3463.81	2.0	0.0115
miR-1246	4118.99	271.75	16.1	0.02431
miR-574-5p	1984.81	383.66	5.6	0.01881
miR-374b-5p	1113.04	322.55	3.6	0.00283
miR-495-3p	1048.32	537.18	2.0	0.00033
miR-23b-3p	1018.65	290.86	3.6	0.00047
miR-197-3p	658.81	310.78	2.2	0.02496
miR-574-3p	625.27	164.21	4.0	0.00979
miR-34a-5p	575.65	202.6	3.0	0.0171
miR-19b-3p	529.06	261.05	2.0	0.00993
miR-106b-5p	521.13	218.05	2.4	0.00096
miR-214-3p	479.95	171.42	2.8	0.00111
miR-193b-3p	472.47	129.99	3.7	0.01471
miR-1290	432.46	36.65	13	0.0375
miR-30b-5p	400.98	65.07	6.4	0.00734
miR-29b-3p	338.76	130.27	2.6	0.0324
miR-15b-5p	318.36	148.02	2.4	0.04894
miR-320b	294.83	92.36	3.2	0.01741
miR-365a-3p	282.92	127.6	2.5	0.03148
miR-365b-3p	282.92	127.6	2.5	0.03148
miR-487b	204.27	88.43	2.3	0.00437
miR-130a-3p	172.66	84.39	2.1	0.00158
miR-485-5p	170.74	75.44	2.3	0.00023
miR-193a-3p	168.1	17.09	9.8	0.00266
miR-331-3p	166.59	32.65	5.2	0.00393
miR-181d	158.26	57.68	2.8	0.00281
miR-320c	147.98	22.5	6.7	0.01261
miR-664a-3p	123.31	14.54	8.7	0.00022
miR-299-5p	122.54	43.88	2.9	0.00022
miR-132-3p	112.94	47.1	2.5	0.01702
