# Supplementary material for: Osteoblasts secrete miRNA-containing extracellular vesicles that enhance expansion of human umbilical cord blood cells
Source: Sci Rep. 2016 Sep 2;6:32034. doi: 10.1038/srep32034 (PMC5009378; doi:10.1038/srep32034)

Osteoblasts secrete miRNA-containing extracellular vesicles that enhance expansion of human umbilical cord blood cells

Jess Morhayim[1], Jeroen van de Peppel[1], Eric Braakman[2], Elwin W. J. C. Rombouts[2], Mariette N. D. ter Borg[2], Amel Dudakovic[3], Hideki Chiba[4], Bram C. J. van der Eerden[1], Marc H. Raaijmakers[2], Andre J. van Wijnen[3], Jan J. Cornelissen[2], and Johannes P. van Leeuwen[1,*]

[1]Department of Internal Medicine, and [2]Department of Hematology, Erasmus Medical Center, Rotterdam, the Netherlands, [3]Department of Orthopedic Surgery, Mayo Clinic, Rochester, MN, USA, [4]Fukushima Medical University School of Medicine, Hikarigaoka, 960-1295, Fukushima, Japan

Supplementary Information

Supplementary Figure S1. MA plot (fold change versus EV abundance) showing the expression level of the selectively enriched EV-miRNAs. The most abundant (miR-146a and miR-29a) and the most enriched (miR-1246 and miR-1290) miRNAs are shown in blue.

Supplementary Figure S2. (a-b) Osteoblast-EVs increase the *ex vivo* expansion of (a) TNCs and (b) $CD34^+$ cells after 10 days of expansion with SCF, Flt3L and SR1 compared to control (N = 2). **(c-d)** Osteoblast-EVs increase the *ex vivo* expansion of (c) TNCs and (d) $CD34^+$ cells after 10 days of expansion with SCF, Flt3L and TPO compared to control (N = 2). Expansion is shown as fold change (FC) increase in total cell number compared to input. Empty and full shapes show the different donors. Control denotes the cells cultured in the absence of osteoblast-EVs.

Supplementary Figure S3. The frequencies of human lymphoid and myeloid lineages in the bone marrow 21 weeks post-transplantation (N = 5 mice/ group).

Supplementary Table S1. Primer sequences

	**Forward Sequence (5'->3')**	**Reverse Sequence (5'->3')**
**BCL2**	AGTACCTGAACCGGCACCT	ACAGTTCCACAAAGGCATCC
**CDC42EP2**	GTCCAGCTCCTGAGACCTTG	GCACTTTGGTCTTGTCACCG
**COL1A1**	GACATGTTCAGCTTTGTGGACC	TGATTGGTGGGATGTCTTCGT
**ELN**	TCCCGGGAGTTGGCATTTC	CAAACTGGGCGGCTTTGG
**GAPDH**	CCGCATCTTCTTTTGCGTCG	CCCAATACGACCAAATCCGTTG
**HBP1**	CCTGTGATGAACACATGGAGC	TGGTACATGCCAGATTGGGT
**PTEN**	TGGATTCGACTTAGACTTGACCT	ACGCCTTCAAGTCTTTCTGC
**TET2**	AGCAGCAGCCAATAGGACAT	TTCCATCAGGCTTGCTTCGG

Supplementary Figure S1 (van Leeuwen)

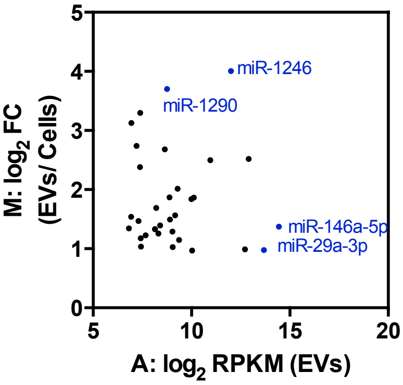

Supplementary Figure S2 (van Leeuwen)

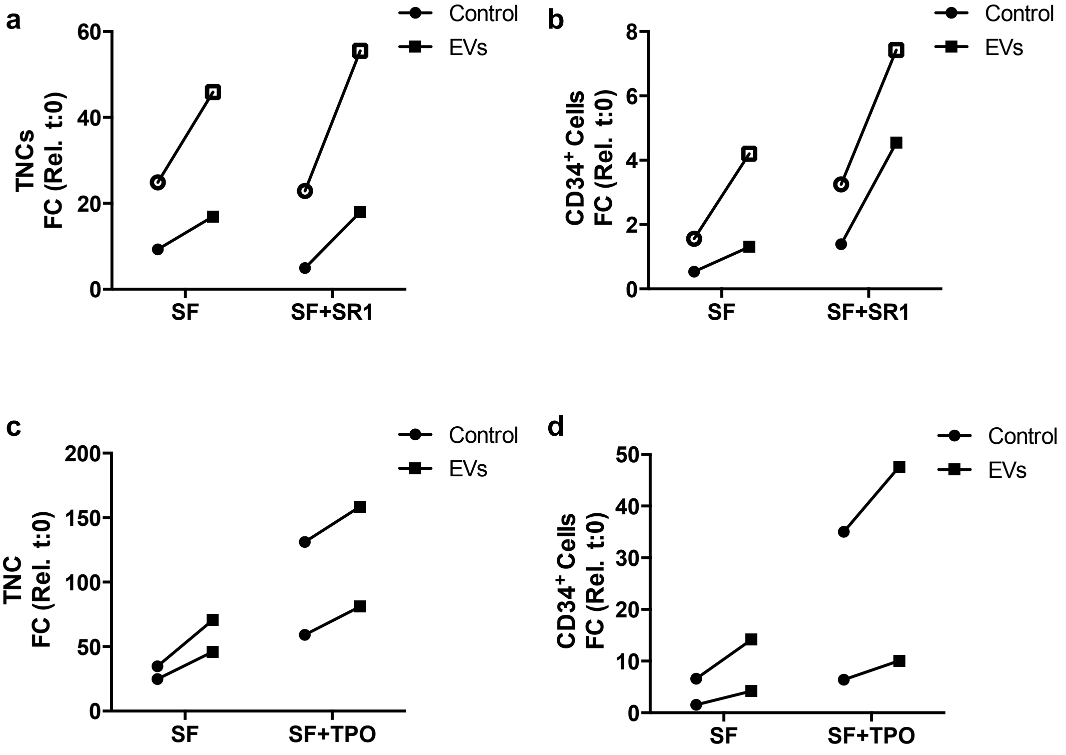

Supplementary Figure S3 (van Leeuwen)

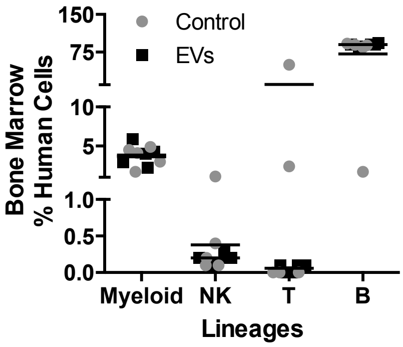

Supplement: Supplementary Information [file srep32034-s1.pdf]
